# Changes in Emotional and Behavioral Problems Between 2000 and 2011 Among 16-Year-Old Polish Children: A Cross-Sectional Study

**DOI:** 10.1007/s10578-018-0791-y

**Published:** 2018-03-05

**Authors:** Ł. Konowałek, T. Wolanczyk

**Affiliations:** Department of Child Psychiatry, Public Paediatric Teaching Hospital of Medical University of Warsaw, Klinika Psychiatrii SPDSK, ul. Żwirki i Wigury 63A, 02-091 Warsaw, Poland

**Keywords:** YSR, Adolescents, Time related changes, Poland, Internalizing

## Abstract

Since after the second world war there has been an increasing number of studies investigating secular changes in adolescent mental health. Although no general trends could be outlined, the majority of studies show at least partial deterioration of psychological wellbeing from year 2000 on. Our study adds to this knowledge by exploring changes in self-declared emotional and behavioral problems in Poland, which is a part of post-communist Europe. In this paper, we compared responses on the Youth Self-Report by Polish 16-year-olds from 2000 and those from 2011. Two independent samples consisted of 259 (year 2000) and 185 (year 2011) 16-year-olds of both genders, drawn from randomized, normative, school-based groups. We analyzed linear, ordinal and binary logistic regression models. The results revealed that teenagers from 2011 reported more self-rated internalizing and total problems. Social and thought problems also rose significantly. Gender related time trends hint at a male increase in externalizing, aggressive behaviors and anxiety/depression. Caseness rose significantly in most scales with female gender being an additional risk factor for internalizing and total problems. No reduction in self-reported emotional and behavioral problems was detected.

## Introduction

Keeping track of changes in self-rated emotional and behavioral problems in adolescents is an important source of information about their psychological condition in a progressing society. Cross sectional studies, among others, help to determine the threshold for norm and pathology over generations, which is a prerequisite for a valid diagnosis. Moreover, they reflect the influence of social, political and economic changes on adolescent psychological well-being. Policy makers and education professionals can use these studies to learn which factors contribute to emotional and behavioral problems of teenagers and which do not. Beyond this, they get a piece of first-hand information from the youth themselves, which we know can differ substantially from parent and teacher observation [1]. Addressing the right causes may reduce the prevalence of depression, anxiety disorders, conducts disorders, etc., thus lowering the cost of treatment and incarceration. In 1995, a review [2] of existing data from the 1950s–1980s demonstrated that psychological problems of the youth in Western countries increased steadily. Hypothetical reasons included: increased unemployment, changes in family structure and functioning, increased life expectancy and better economic conditions. Also, the advent of mass media was discussed. Maughan et al. [3] readdressed this issue by inspecting data from the 1980s–2000s. They concluded that, although many self-report studies showed significant increase in emotional and behavioral problems “reasons for these changes remain elusive”.

We reanalyzed papers included in Maughan et al. [3], which cover countries like: the U.S. [4], Greece [5], the United Kingdom [6–8] and Sweden [9]. Moreover we added data from the U.S. [10], and The Netherlands [11]. Only studies using self-report measures were selected. In the U.S. studies seem to contradict each other. While Achenbach et al. [4] found evidence of small but significant improvement in self-reported psychological well-being, Twenge et al. [10] reported that “adolescent mental health has declined significantly since the 1980s,” until 2002. Both studies covered a similar period (1989–1999 for Achenbach’s study [4] and 1985–2002 for Tewnge’s study [10]) but used different measures (the Youth Self-Report (YSR) in the Achenbach’s study [4] and Minnesota Multiphasic Personality Inventory (MMPI) in the study by Twenge et al. [10]). The Greek study (1980–1998) [5] used The General Health Questionnaire (GHQ-28) and reported significant increase in mental health problems and some sub-scales (somatic complaints, anxiety, social dysfunctions and depression) with no effects of gender. The authors also reported a disappearing effect of selective migration. In the U.K., studies differ in England [6, 7] and in Scotland [8], although both used the same measure—the 12 item General Health Questionnaire (GHQ-12). In England (Collishaw (2004) [6]: (1979–1999), Collishaw (2010) [7]:(1986–2006)) researchers found increases in self-reported conduct and emotional problems (namely anxiety and depression) with no effects of social status, gender, changes in family structure or ethnic composition. The Scottish study (1987–2006) [8] reported increased caseness (scoring over a cut-off point on a questionnaire) for girls (from 19 to 33%), but not for boys (13–15%). Girls also seemed to be more affected by school performance worries. No gender differences were found for personal worries (e.g. looks, weight). In Sweden (1970–1996) [9] girls reported more anti-social and self-esteem problems but also better peer relations on an original Symptom Questionnaire. Other domains (e.g. relations with parents, teachers, physical and emotional problems) remained stable. Boys were not assessed. Finally, in The Netherlands (1993–2003) [11] small changes were detected as measured by the YSR. Boys reported decreases on behavioral scales while girls showed worsening of internalizing (e.g. somatic complaints, depressive symptoms) and social problems. Interaction effects of gender, socioeconomic status (SES), parent education and age were discovered for somatic complaints (decreased in older boys only), drug abuse (increased in boys from families with low parental education and occupation), aggressive behaviors (increase in younger girls and decrease in older ones) and total score (increase only in younger adolescent girls).

For the next period (from the year 2000 until now), which will be the focus of this paper, we will look at results from The Netherlands [12], Finland [13, 14], Germany [15], Norway [16], Scotland [17] and Iceland [18]. The Dutch research [12] showed no persistent change in behavioral and emotional problems of Dutch youth across 2003–2013, as measured on the 25-item Strengths and Difficulties Questionnaire (SDQ). Some trends from this study included: higher hyperactivity and also more peer and conduct problems in vocationally trained adolescents (versus academically educated); more conduct problems in ethnic minority group and more hyperactivity in ethnic majority. In Finland [13] time trends (2002–2013) showed a decline in internalizing and externalizing problems among 15–16 year old boys. Among others, the YSR was used. A somewhat earlier (1998–2006) study from Finland [14] showed no changes in emotional and behavioral problems. However, a decrease in prosocial behavior among girls was detected. The 1998–2006 study used a different methodology (SDQ) and a wider age span: 13–17 years. The German study [15], using Kidscreen10 (a 10-item questionnaire) and SDQ, found no changes for 11, 13 and 15 year olds between 2006 and 2011. In Scotland [17], between 1994 and 2006: “significant increases over time were observed for all mental well-being measures among girls and for all but confidence among boys.” Time trends also demonstrated an emergence of socioeconomic inequalities in young people’s happiness and confidence. Data was collected with the use of the Health Behavior in School-aged Children (HBSC) survey, containing four mental well-being items and a short list of health complaints to choose from. Finally, in Iceland [18] (1997–2006), a study of 14–15 year olds, using a 14-item questionnaire concerning depressive and anxiety symptoms, produced results showing increased levels of anxiety symptoms in general and a rise in depression levels among girls.

We also found data from Sweden [19] covering a broad time period (1988–2011). The respondents were 15–16 year old boys and girls and the measurement tool was the 8-item Psychosomatic Problems questionnaire. The general conclusion was that adolescent mental health had gotten much worse in the 1990s, compared to the preceding decade. However, as authors claim: “This trend has leveled off and is now down going.” A Norwegian study [20] covers a similar period (1992–2010). Here, the Depressive Mood Inventory (6 items) was filled in by 16–17 year olds at multiple points in time. “The results showed that the prevalence of high scores on depressive symptoms increased significantly between 1992 and 2002 among both boys and girls. No significant changes were observed between 2002 and 2010.”

In conclusion, 12 out of 17 studies [5–12, 14, 16, 18, 19] reported at least partial worsening of adolescent psychological well-being and/or behavioral problems. Seven studies reported decreases in emotional and behavioral problems [3, 4, 10, 11, 13, 17, 19], one reported no changes at all [15] and three traced multiple time fluctuations [9, 12, 19]. It is possible that this varied outcome was at least in part due to the methodology used. Studies using short, psychiatry oriented forms (like the GHQ) seem to show more decline in psychological well-being than longer questionnaires covering more domains of emotional and behavioral troubles (like the YSR or the MMPI).

Gender specific trends were observed in Scotland [8, 17], The Netherlands [11], Finland [13, 14], Iceland [18] and Sweden [19]. Other studies reported no gender differences. In every case girls reported more problems, with the exception of Scotland [17]. Also, in Scotland boys experienced no increases in confidence, as opposed to girls. In most cases, girls are more troubled with internalizing difficulties (anxiety and depression). However, in The Netherlands [11], Finland [14] and Sweden [19] a female rise in externalizing (aggressive and antisocial behaviors) was observed. Surprisingly, no studies reported trends linked to SES, with the exception of The Netherlands [12], where drug abuse increased in children from underprivileged families and Scotland [17], where SES started affecting adolescents’ happiness and confidence in 2006.

To our knowledge, there has been no research concerning time related trends in psychological problems of the youth in post-communist countries of Central and Eastern Europe. These countries underwent a massive transformation in the 1990s which entailed a significant decline in SES of many families (for Polish data consult the Central Statistical Office, GUS [20]).

Police statistics [20] show that the number of underaged criminals had increased from 76,442 in 2000 to 101,026 in 2011, suggesting a rise in juvenile delinquency and aggression. The 2000s were also a period of a major education reform. Time spent in elementary school was shortened from 8 to 6 years. A new stage of education, intermediate between elementary and upper secondary schools, was introduced (“gimnazjum”). Additionally, central examinations at the end of every stage were initiated, increasing school pressure and demands.

In light of the above mentioned facts we conclude that the years 2000–2011 in Poland had been a period of economic growth and, on the other hand, increased educational pressure. Other studies [7, 8] highlighted the role of educational demands on adolescent emotional and behavioral problems. In Poland, SES is highly connected to urbanization—bigger cities tend to concentrate inhabitants with higher income. It would be interesting to know whether this plays a role in self-reported psychological well-being of the youth. What this study set out to investigate were the 11-year changes (2000–2011) in self-reported emotional and behavioral problems of Polish 16-year-olds in the general population. We checked for gender differences, urbanization and interactions. The second aim of this study was to examine whether these potential changes resulted in higher caseness and thus a higher need for psychological and psychiatric attention.

## Method

### Study Design

This study consists of two independent stages of assessments with a time interval of 11 years. The study design is cross-sectional, meaning that each subject is assessed only once. This allows for evaluation of between subject factors (year of study, sex and urbanization). Gender and year of study were assessed on two levels (male vs. female; 2000 vs. 2011), while urbanization was evaluated on a four level scale. Urbanization for the 2011 sample was included in the sociodemographic survey. In it, respondents had to choose between: village; small town; medium town; large town or suburbia. In the 2000 sample urbanization was determined based on the size of the school settlement. Selection criteria were in accordance with GUS classification (see Table 1).

**Table 1:** Levels of urbanization categorization algorithm (self-declared in 2000)

Number of inhabitants	Categorization	Participants from 2000	Participants from 2011
< 5000	Village (1)	26	17
5000–20,000	Small town (2)	26	110
20,000–200,000	Medium town (3)	103	40
> 200,000	Large city/suburbia (4)	104	18

Only 16-year-olds, that is people who were after their 16th birthday and before their 17th birthday on the day of assessment, were included in our analysis. This was done solely for practical reasons, as during the second stage of assessment only 14, 15 and 16-year-olds were asked to fill the questionnaire. Moreover, 16-year-olds made up over 90% of the study group. Therefore we decided to omit the other age groups as there were not enough respondents to provide reliable results.

### 2000 Sample

The group from 2000 was extracted from a previous publication on standardization of the Polish version of ASEBA [21]. That sample, counting 3132 children in general, had been drawn from a school-attending population of Polish 7–19 year-olds. The scholarisation index for Poland at that time was 99.3% of the elementary school population and 90.4% of the secondary school population [22], meaning that only a small percentage of youth were excluded by this procedure.

Schools were selected from an address list provided by the Ministry of Education. The list contained all types of schools except for special facilities for children with intellectual disabilities. Random stratified sampling was used. Strata included location, level of education and urbanization. For more details see the original thesis [21]. All questionnaires were filled between September 1999 and March 2000. A letter was sent to each of the selected schools, informing about the purpose of the study and asking for participation. A letter from the Ministry of Education, supporting the study was attached. A researcher responsible for the school’s region would then call the school principal and select one of the parallel grades using a random numbers table. Next, the class tutor was consulted by the researcher to learn about the upcoming school reunion date and to hand in the Teacher Report Form (TRF) and YSR questionnaires. Child Behavioral Check List (CBCL) questionnaires were filled in by parents during school reunions. Parents were informed that filling in the CBCL was equivalent to giving consent to their child’s participation in the study. TRFs were filled in by class tutors. YSRs were distributed to the youth by class tutors and were filled in during class. Tutors were paid ten PLN for each TRF as a compensation for their time.

323 children were aged 16 and only they were included in the present analysis. We also applied an inclusion criterion for at least 96 problem items (96%). This eliminated another 64 (18%) of the respondents. For the remaining 259 respondents, missing values were replaced by a median of the neighboring 10 responses (5 before and 5 after the missing value).

### 2011 Sample

The remaining participants came from a study carried out by “Nobody’s Children Foundation” (http://fdn.pl/) in May 2011. This time only third grades of secondary schools (gimnazjum) were taken into consideration and only YSRs were administered. 12 Schools were selected by random sampling, all of which agreed to participate. Schools came from 12 different regions (out of a possible 16). Schools were approached in the same manner as in the previously mentioned study. YSRs were also administrated by teachers during class. Apart from the YSR, other measures were administered: a sociodemographic survey, and a number of questionnaires on internet using habits. Students who agreed to take part in the study received educational materials on safe internet usage. Total sample counted 235 adolescents aged 15–18. However, 212 students (90.2%) were aged 16. To avoid any confounding effects of age, only 16 year-olds were included in the present study. After applying the inclusion criterion of 96 ticked items, 185 participants were selected for the present analysis. Again, missing items were substituted by a median of ten neighboring values.

### The Youth Self-Report (YSR)

The YSR is a self-assessment questionnaire, composed of 119 problem items and 7 competence items. 16 of the problem items refer to socially desirable qualities. Items were rated 0 = not true, 1 = somewhat or sometimes true and 2 = very true or often true, on the basis of the preceding 6 months. Items 2 and 4 (questions about asthma and allergy) are not included in any of the scale scores [23].To sum up, only 101 problem items were taken into consideration, as competence scales demonstrated very low reliability in the Polish standardization [21]. Problem items make up eight syndrome scales: withdrawn, anxious/depressed, somatic complaints, social problems, thought problems, attention problems, delinquent behaviors, aggressive behaviors, which in turn compose internalizing and externalizing broad band scales. Additionally, a total problems score is calculated by summing all problem items. Syndrome scales were developed empirically through factor analysis.

The Polish version was translated and adapted by Wolanczyk [21]. It showed good reliability: Cronbach’s alpha coefficients range from 0.62 for social problems to 0.95 for total problems (M = 0.78; SD = 0.11). In our sample, Cronbach’s alpha coefficients ranged from 0.63 for social problems to 0.96 for total problems (M = 0.81; SD = 0.10). Validity was measured as the power to discriminate between children with and without a psychiatric diagnosis. Also intercorrelations between subscales and correlations with other measures (CBCL and TRF) were measured as part of validity testing. Children with a diagnosis had significantly higher scores on the YSR (except for delinquent behaviors and aggressive behaviors). Correlations with CBCL and TRF were lower than in other countries. Intercorrelations between scales were significant but low, which proved satisfactory theoretical validity. Also the eight syndrome structure was confirmed, as demonstrated by Ivanova et al. [24].

This questionnaire was chosen because it allows for both general and symptom specific evaluation of psychological wellbeing in adolescents. It is also the only questionnaire fully adapted to Polish conditions.

### Data Analysis

The data was analyzed using IBM SPSS Statistics (Version 21). All scale results were highly skewed. Therefore we could not perform linear regression models (LRM) on the raw scores. We square-root-transformed the raw scores on the broad-band scales (internalizing, externalizing and total problems). This produced normal distributions of the residuals according to Shapiro–Wilk tests (*p* ≥ .32). We then applied the LRM to measure associations between such scores and year of study, gender, urbanization and any interactions thereof.

We did not manage to achieve normal distributions of the residuals of the eight problem scales through square-root-transformation. We therefore decided to evaluate the influence of year of study, gender, urbanization and interactions on odds of getting a higher score through ordinal logistic regression (OLR). As distances between points on the scales are not relevant to OLR, we ranked the scale scores into seven percentile groups (or thresholds) This procedure allowed us to reduce the number of empty cells. The number of groups was set at seven because in every scale more than half of the respondents scored seven or less. This resulted in the creation of new ordinal variables, which we later introduced into the OLR models. Results are given as *β* parameters and 95% confidence intervals (CI). A *β* can be interpreted as the increase in either the square root of a scale score (in LRM), or the log odds of crossing any particular threshold on the scale (in OLR, see formula below), given a single unit increase in the predictor.

$$\ln \left[ {\frac{{{\text{p}}\left( {{\text{x}} \leq {\text{m}}} \right)}}{{1 - {\text{p}}\left( {{\text{x}} \leq {\text{m}}} \right)}}} \right]={\uptau _{\text{m}}} - \upbeta {\text{X}}$$

where *τ*_*m*_ represents threshold values for each threshold *m*; **β** is a vector of regression coefficients; **X** is a vector of values of independent variables.

Interactions were measured by multiplying independent variables (e.g. “year of study” × “gender”) and introducing them to the model as separate variables.

T scores were used to analyze clinical intervals. We followed Wolanczyk’s [21] suggestion and set two clinical intervals: 60–100 T for internalizing, externalizing and total score scales; and 67–100 T for syndrome scales. Wald stepwise backward entry binary logistic regression was applied to determine significant predictors of adherence to a clinical group. Gender, year of study, urbanization and interactions were introduced as predictors. Inclusion *p* value was set at *p* = .005, exclusion at *p* = .006.

A Bonferroni correction of *p* values for multiple tests was applied. Significance level was set at *p* ≤ .05. For the three LRMs the corrected *p* value was ≤ .018, for the eight OLRs it was *p* ≤ .007 and for the 11 logistic regression models the value was *p* ≤ .005.

## Results

### Structure of Respondent Population

The respondent population structure is presented in Tables 2 and 3. Urbanization groups were not equal in size and urbanization structure was not the same across years of study or gender groups. Table 3 shows that there was no gender disbalance in the total sample nor was there a significant change of gender distribution across samples. There were significantly more respondents from 2000, *χ*^2^ (1,444) = 12.33, *p* < .001.

**Table 2:** Demographic statistics of the sample (% of observations)

	Urbanization			
	1	2	3	4
Year of study^a^				
2000	26 (5.86)	26 (5.86)	103 (23.20)	104 (23.42)
2011	17 (3.83)	110 (24.77)	40 (9.01)	18 (4.05)
Gender				
Male	9 (2.03)	101 (22.75)	51 (11.49)	68 (15.32)
Female	34 (7.66)	35 (7.88)	92 (20.72)	54 (12.16)
Total	43 (9.68)	136 (30.63)	143 (32.21)	122 (27.48)

*χ*^2^ tests were performed to check for equality of frequency distributions across urbanization levels between years of study, gender groups and on their own

^a^*p* < .001

**Table 3:** Demographic statistics of the samples (% of observations)

	Gender^b^		
	Male	Female	
Year of study^a^			
2000	140 (31.53)	119 (26.80)	259 (58.33)
2011	86 (19.37)	99 (22.30)	185 (41.67)
Total	226 (50.90)	218 (49.10)	444 (100)

*χ*^2^ tests were performed to check for equality of gender frequency distributions between years of study and on their own

^a^*p* = .116, ^b^*p* = .704

### Effects of Year of Study and Interactions

We found a significant positive association for year of study with all broadband (internalizing, externalizing and total problems) scale scores (square-root-transformed) as well as with log odds of a higher score on anxious/depressed, social problems, thought problems and aggressive behaviors. This implies that adolescents’ self*-*reported problems increased with time.

The analysis also revealed significant year of study by gender interactions for anxious/depressed, aggressive behaviors and externalizing. The negative coefficient values indicate that female gender was a protective factor against time-related increase on these scales. No effects of urbanization were detected.

Binary logistic regression analysis gave a significant main effect of year of study for somatic complaints, thought problems and internalizing, meaning that respondents from 2011 were more likely to reach a clinical interval on these scales. Year of study by gender interaction effects were significant for internalizing and total problems, meaning that girls were more likely than boys to fall within the clinical range in 2011. Interaction of year of study and urbanization was significant for social problems, which implies that respondents from larger cities had more clinical social difficulties after 11 years. Finally, three-way interactions of year of study, gender and urbanization were yielded for anxious/depressed, Thought problems and delinquent behaviors. A result which indicates that girls from larger settlements would have a higher chance of being considered as clinical cases on these scales in 2011.

Significant *β*s and ORs for main effects of year of study are presented in Table 4.

**Table 4:** Associations between predictors and YSR scales (n = 444)

	W	SC	AD	SP	TP	AP	DB	AB	I	E	TPS
Predictors of higher scores											
Year of study	–	–	1.06 (0.49–1.62)	1.23 (0.66–1.79)	0.93 (0.35–1.50)	–	–	0.91 (0.35–1.47)	0.70 (0.27–1.14)	0.56 (0.16–.96)	1.15 (0.48–1.82)
Year × gender	–	–	− 1.03 (− 1.78 to − 0.28)	–	–	–	–	− 1.17 (− 1.92 to − 0.42)	–	− 0.67 (− 1.20 to − 0.13)	–
Year × urbanization	–	–	–	–	–	–	–	–	–	–	–
Year × gender × urb	–	–	–	–	–	–	–	–	–	–	–
R square^a^	.03	.05	.05	.07	.03	.02	.02	.03	.05	.02	.03
Predictors of clinical adherence											
Year of study	–	2.82 (1.57–5.07)	–	–	3.57 (1.97–6.48)	–	–	–	2.32 (1.47–3.65)	–	–
Year × gender	–	–	–	–	–	–	–	–	0.59 (0.44–.99)	–	2.87 (1.39–5.90)
Year × urbanization	–	–	–	1.58 (1.23–2.03)	–	–	–	–	–	–	–
Year × gender × urbanization	–	–	0.64 (0.73–.87)	–	0.77 (0.65–.86)	–	0.76 (0.66–.88)	–	–	–	–
R square^a^	.00	.05	.08	.07	.11	.00	.06	.00	.07	.00	.06

Predictors of higher scores are presented in the form of β parameters (95% CI) derived from either the OLR (eight problems scales) or the LRM (three broadband scales). Predictors of clinical adherence are presented in the form of odd ratios (95% CI) derived from logistic regression analysis

*W* withdrawn, *SC* somatic complaints, *AD* anxious/depressed, *SP* social problems, *TP* thought problems, *AP* attention problems, *DB* delinquent behaviors, *AB* aggressive behaviors, *I* internalizing, *E* externalizing, *TPS* total problem score

^a^Nagelkerke R square in OLR and binary logistic regression models

## Discussion

### Overview

This study’s aim was to scrutinize how Polish 16-year-olds viewed their psychological condition in 2011 in contrast to that from 2000. The general conclusion is that after 11 years Polish 16-year-olds reported more behavioral and emotional difficulties on most scales.

We did not observe any effects of urbanization on probabilities of a higher score. This could be a more universal phenomenon—also in England [6, 7] no effects of SES were reported. Perhaps social factors do not play a significant role in emotional and behavioral well-being of adolescents. On the other hand, Dutch [11] and Scottish [17] studies did show some SES interactions. Our findings in this matter should be treated with caution as we did not manage to maintain equal distributions of urbanization over time.

### Time Related Changes

The effect of year of study was the biggest on social problems. This is mainly because the median score in 2000 was unusually low (1) compared to other age groups. This is not easy to explain, as there is no obvious reason why 16 year-olds and not 15 or 17 year-olds should report much less social problems. The only, to our knowledge, global social change that affected all youth in this period was the education system reform. In 2011 16-year-olds were freshmen to high-school (“liceum”,“technikum” or “szkoła zawodowa”), which was not the case in 2000 when they would usually have had one full year of high-school behind them. Maybe in 2000 our respondents became well-adjusted to their new peer groups and could sacrifice more time to social bonding rather than to their final exams preparations (2 years ahead). Compared to other studies, our findings are unique in this regard. Tick, van der Ende and Verhulst [11] reported a small increase in girls’ social problems but overall the score lowered with time. Also, in Sweden [9] girls reported better peer relations.

Thought problems also rose significantly. This parallels a small increase with regard to these problems found in Dutch youth between 1993 and 2003 [11].

Lastly, there was a significant year of study effect on internalizing. This is interesting because there was no such effect on any of its component scales (with the exception of a gender moderated effect on anxious/depressed). This means that relatively small, statistically insignificant increases on other scales (withdrawn and somatic complaints) amassed to give this result. This could be an important clinical finding, as clinicians, teachers, parents, etc. should pay attention to a generalized tendency toward internalizing rather than look for specific symptoms.

### Moderating Effects of Gender

In line with our initial suppositions, we did observe an increase in aggressive behaviors and externalizing (but not in delinquent behaviors). Interestingly, this seems to be a gender related phenomenon as female gender greatly reduces (or even removes) the impact of time-related changes. This entails that boys became more aggressive after 11 years. Surprisingly, this seems to be a uniquely Polish phenomenon, as other studies show either no gender related changes or an increase in female aggression (Sweden [9], The Netherlands [11] and Finland [14]).

Although no significant differences in internalizing were detected, we did observe a male-specific increase in anxiety/depressive symptoms. This proves that after 11 years boys experienced more emotional pressure which was dealt with mainly through increased externalizing symptoms.

### Clinical Adherence

Even though emotional and behavioral self-reported well-being of adolescents does seem to get worse in many instances, these results do not automatically reflect what mental health care clinicians could observe. Adolescents from 2011 were approximately three times more likely to reach a clinical interval in thought problems and internalizing, which is consistent with previous discussion. Interestingly, although our OLR analysis did not yield a significant association of year of study and somatic complaints score, respondents from 2011 were about three times more likely to be included in the clinical group. This suggests that a relatively small group of “high somatizers” appeared in 2011.

Moreover, although in general there were few gender differences, and the ones that did appear were unfavorable for boys (more anxiety–depressive symptoms and aggression), the clinical group was actually more populated by girls after 11 years. This is true for internalizing and total problems. Interactions of year of study, gender and urbanization were significant for anxious/depressed, thought problems and delinquent behaviors, meaning that girls from larger towns were more likely to get a clinically significant score in 2011.

Changes in clinical intensity of social problems were proportional to the level of urbanization.

In general, caseness rose quite substantially (although not always statistically significantly) on all scales (Table 5). This is troubling because many more adolescents need some form of psychological or psychiatric support. On the other hand, it may be necessary to revise Polish clinical thresholds. This holds especially for externalizing where > 42% of respondents from 2011 were included in the clinical group.

**Table 5:** Percentages of respondents falling into a clinical range

	% within the clinical range 2000	% within the clinical range 2011
Withdrawn	4.30	10.80
Somatic complaints	7.80	19.50
Anxious/depressed	10.90	18.90
Social problems	2.30	11.90
Thought problems	13.50	28.10
Attention problems	3.50	6.50
Delinquent behaviors	15.20	23.80
Aggressive behaviors	5.40	9.70
Internalizing	19.50	31.90
Externalizing	37.80	41.60
Total problems	14.40	27.02

### Comparison to Other Studies

Our study adds to the body of knowledge on self-reported emotional and behavioral problems among adolescents in Europe. Our results show a general decline in Polish 16 year olds’ emotional and behavioral well-being, which is consistent with findings from Greece [7] and Sweden [19] in the 1990s. In Sweden, adolescents got “better” in the following decade. It would be interesting to see if the situation in Poland will be similar.

Another similar finding is an increase in thought problems (The Netherlands [11]).

What seems to be unique for our sample is the general increase in social problems (although the “starting point” in 2000 was very low) and an increase in male aggression and externalizing. No other study reported a general increase in internalizing, although it is important to note that many psychometric tools employed in them did no measure it directly.

In parallel to Scottish results [8], we found caseness to be greater in girls rather than boys (although many gender unrelated effects exist).

### Strengths and Limitations

This study’s most central strength is its usage of the same, directly comparable and psychometrically sound measurement of self-declared emotional and behavioral difficulties. Maughan et al. [3] pointed out that this has often not been the case for comparative cross-sectional studies.

Also, effort was made to include a wide scope of locations, school types and balanced gender proportions. This makes the results generalizable to the wider population of Polish school-attending 16-year-olds.

One of the main limitations is that our study is restricted to 16-year-olds. Wider age span would allow us to determine whether observed social difficulties are merely an adaptive reaction to a change of school environment or a broader phenomenon.

On a different note, Rescorla et al. [25] found that gender, age and location (on a country level) and their interactions accounted for < 10% of total variation of YSR scores—a result which is reproduced in this study. This suggests the future use of other variables, such as temperament, number of close friends, relations with caregivers etc.

Lastly, urbanization was evaluated differently in both samples. One method relied on self-report and the other on objective data. Both have their advantages. Self-report is superior in that it permits individual evaluation (students from the same class may come from different settlements). Objective measurement, on the other hand, is immune to personal bias in evaluating one’s settlement’s size. Although we believe that both measurements were comparable, we cannot exclude the possibility that different methodologies led to imprecision and false results.

Further research should include different age groups and more explanatory variables to provide a bigger picture of the adolescents’ psychological condition. It would also be helpful to conduct research in smaller time intervals.

## Summary

The present study revealed a trend towards deterioration of the self-reported emotional and behavioral conditions of Polish 16 year olds between 2000 and 2011. Main changes include higher internalizing, externalizing and total problems scores, as well as a bigger likelihood of a higher score on social and thought problems in 2011. Gender related trends were detected with boys declaring more externalizing and anxiety/depression related symptom after 11 years. Changes in clinical adherence appear to concern girls more than boys, especially in terms of internalizing and total problems. Girls from larger towns seem to be more affected by thought problems, delinquency and anxiety/depression. Changes in clinical intensity of social problems seem to trouble adolescents from larger settlements.

These results show similarities and differences to other European samples. Further cross-sectional and longitudinal studies of Polish adolescents are needed.
